# Evaluation of demographic and co-infection pattern amongst HIV seropositive individuals in a tertiary care centre

**DOI:** 10.6026/973206300221469

**Published:** 2026-03-31

**Authors:** Madhuri Gattani, Manish Purohit, Ashish Kumar Joshi

**Affiliations:** 1 Department of Microbiology, Laxminarayan Pandey Government Medical, Ratlam, Madhya Pradesh, India; 2 Department of Microbiology, MGM Medical College, Indore, Madhya Pradesh, India

**Keywords:** Human immunodeficiency virus (HIV), demographic profile, co-infections, tuberculosis (TB), tertiary care centre

## Abstract

HIV infection remains a major public health issue in India, with demographic characteristics and co-infections influencing disease progression. Therefore, it is of interest to evaluate the demographic profile and co-infection patterns among HIV-positive individuals attending a tertiary care centre. The research involved 110 participants, analyzing factors such as age, gender, mode of transmission and co-infections. The results show that young, sexually active males are most affected, with tuberculosis being the leading co-infection. This study advances our understanding of HIV co-infection patterns, emphasizing the importance of early screening and integrated management for improved outcomes.

## Background:

Human immunodeficiency virus (HIV) infection is still a major global health concern, especially in low- and middle-income nations. Despite great breakthroughs in antiretroviral therapy (ART), HIV remains a major issue due to its chronic nature and relationship with opportunistic and co-infections [1]. India has the world's second-highest HIV load, accounting for a significant portion of the overall number of individuals living with HIV [2]. HIV epidemiology in India is diverse, impacted by socioeconomic factors such as age, gender, marital status and behavioral habits [3]. Young individuals in the reproductive and economically productive age groups are disproportionately affected, with serious social and economic repercussions. Sexual transmission is the most common way of HIV acquisition in India; however mother-to-child transmission and blood-borne routes continue to contribute to new infections [4, 5]. Co-infections with tuberculosis (TB), hepatitis B virus (HBV), hepatitis C virus (HCV) and syphilis are frequent in PLHIV due to shared transmission pathways and HIV-induced immunosuppression [6]. These co-infections complicate clinical care, raise morbidity and mortality and impair treatment results [7]. Tuberculosis, in particular, is the most frequent opportunistic infection among HIV-positive people in India [8]. Understanding the demographic features and co-infection patterns of HIV seropositive people is critical for developing focused preventative interventions and providing optimal patient care [9]. Therefore, it is of interest to describe the demographic profile and co-infection patterns of HIV-positive individuals attending a tertiary care center to inform targeted interventions and improve patient care.

## Methodology:

This prospective observational study was conducted in the Department of Microbiology in collaboration with the ART Centre, MGM Medical College and associated Hospital, Indore, over a period of one year from 06 December 2022 to 05 December 2023. The inclusion criteria were confirmed HIV seropositive individuals aged ≥1 year, patients registered at the ART centre and receiving ART, patients who provided informed written consent (or assent with guardian consent where applicable) and patients willing to participate and comply with study procedures. Exclusion criteria included HIV seronegative individuals, patients unwilling or unable to provide informed consent, patients with incomplete clinical or laboratory records relevant to the study and patients lost to follow-up during the study period.

## Results:

The study included 110 confirmed HIV seropositive individuals who were registered and receiving antiretroviral therapy (ART) at the ART centre during the study period. Majority of participants belonged to the 21-40 years' age group (67.27%), followed by 41-60 years (21.81%), 1-20 years (10%) and >60 years (0.9%). The mean age was 32.58 ± 11.62 years. Out of 110 study subjects, 81 (73.63%) were males, 28 (25.45%) were females and 1 (0.9%) was transgender. Tuberculosis was the most common co-infection (21.82%), followed by syphilis (7.27%), hepatitis B (4.55%) and hepatitis C (4.55%). Multiple co-infections were also observed. Table 1 (see PDF) shows the age distribution among study subjects, with the majority (67.27%) falling in the 21-40 years age group, followed by 41-60 years (21.81%), 1-20 years (10%), and >60 years (0.9%). Table 2 (see PDF) highlights the gender distribution, with 73.63% males, 25.45% females, and 0.9% transgender participants. Figure 1 (see PDF) depicts the distribution pattern of the mode of transmission among study subjects. Table 3 (see PDF) presents the HIV distribution with or without co-infection, showing that 32.72% had co-infections. Table 4 (see PDF) provides a detailed distribution of co-infections, indicating the presence of various co-infections such as tuberculosis (21.82%), syphilis (7.27%), hepatitis B (4.55%), and hepatitis C (4.55%), with some subjects having multiple co-infections. Table 5 (see PDF) summarizes the WHO clinical staging at diagnosis, with the majority of participants diagnosed in stages I and II (60.9% and 12.7%, respectively), and fewer in stages III and IV. Finally, Table 6 (see PDF) illustrates the HIV status of the spouse among study subjects, revealing that 37.3% of married participants had HIV-positive spouses, 25.5% had HIV-negative spouses, and 37.3% were not applicable. Data collection involved recording demographic details such as age, gender, mode of transmission and spouse HIV status using a structured proforma. Screening for co-infections including tuberculosis, hepatitis B, hepatitis C and syphilis was performed as per national guidelines and WHO clinical staging at the time of diagnosis was also documented. Statistical analysis was conducted by entering the data into Microsoft Excel and analyzing it using Statistical Package for the Social Sciences (SPSS) software. Descriptive statistics were used to summarize the data and categorical variables were expressed as frequencies and percentages.

## Discussion:

The current study found that HIV infection primarily affects young adults, mainly males, indicating this age group's socio-behavioural vulnerability. Similar demographic patterns have been observed in research conducted in several parts of India and other emerging countries [10]. Sexual transmission was revealed as the most common form of HIV acquisition, in line with national and international assessments that identified heterosexual contact as the key driver of India's HIV pandemic [11]. This highlights the importance of improving sexual health education and supporting safe sexual practices. Approximately one-third of the study population had co-infections, with tuberculosis being the most common. This finding is consistent with numerous studies that have identified tuberculosis as the most common opportunistic illness among people living with HIV due to immunological suppression [12]. The burden of HIV-TB co-infection is a serious problem for public health systems, necessitating integrated HIV-TB services. Co-infections with HBV, HCV and syphilis were also detected, indicating common transmission channels and emphasizing the significance of routine screening for these infections in PLHIV [13]. The prevalence of HIV-positive spouses in a considerable number of participants highlights the importance of couple-based counselling and testing in preventing further transmission [14]. Overall, the findings emphasize the need for early detection, extensive screening for co-infections and integrated care approaches for improving clinical outcomes among HIV seropositive people.

## Conclusion:

In the present study, HIV seropositivity was most prevalent among young, sexually active males. Sexual transmission remained the primary mechanism of acquisition, with tuberculosis being the most common co-infection. Strengthening preventative efforts, early identification and integrated management of co-infections are critical for improving HIV patients' quality of life and outcomes.
